# A Lecture Introductory to the Course of Obstetrics in Jefferson Medical College of Philadelphia

**Published:** 1842-12-10

**Authors:** 


*A Lecture Introductory to the Course of Obstetrics in Jefferson Medical College of Philadelphia.* Delivered November 5, 1842. By C. D. MEIGS, M. D. Published by the Class. Philadelphia: 8vo. pp. 31.

Professor MEIGS' Introductory presents a very just and candid picture of the profession of a practitioner of midwifery—a faithful representation of the difficulties and responsibilities of this most arduous of callings, vividly, but very calmly and justly drawn. The professor's experience in the practice of midwifery, ample as has been his share of the honours and emoluments of his profession, is far from encouraging to those of his juniors who may turn their eyes to this speciality. He represents it as a bondage from which there is no release, a toil without respite, hardly allowing him time to rear his own children—in the multiplicity of its calls often compelling him to postpone even his daily professional duties to the long detention of some engrossing case. We remember, some time ago, having had occasion to invoke the services of Dr. Meigs in consultation, hearing from him that the night on which we met was the eighteenth in succession that he had been summoned from his repose! In this introductory, Dr. Meigs recounts a recent conversation with "a gentleman, once extensively engaged in obstetric business here."

"I saw," says Dr. Meigs, "that he looked sick and weary. 'I am done with the practice,' said he, 'I have lost my health at it, and I have given it up. I will never engage in it again.' 'Tell me,' said I, 'did you not find it an agitating pursuit?' 'Agitating,' said he, 'I have a disease of the heart—and expect to die suddenly, perhaps soon. An agitating pursuit! I'll tell you what—there is no earthly consideration that could lead me to go through it again, as I have once. Let me see, no! no! short of my eternal salvation, I deem no compensation equal to its demands.'"

The task of mental exertion imposed upon the student of midwifery the professor justly represents to his class as one of the heaviest in medicine.

"Midwifery has become a science as well as an art. It comprises within its limits a vast amount of anatomical and physiological acquisitions, an immense range of therapeutical applications, much of what might be called pure surgery, and is, in fact, a superstructure on the general base of the other medical sciences. It demands all that the general practitioner ever attains to, and beyond that, much that is peculiar and appropriate to its own sphere. Its theories on reproduction, and its deep researches into the arcana of the operations disclosed only by the microscope, or to the eye of reason, are among the most curious and important that can engage the attention of the philosopher. And these researches are not limited in their results to the gratification of a mere idle curiosity, but they are instituted for, and they lead to, the detection of the nature and principles of those affections, and motions, and diseases, which we are bound to comprehend, in order that we may the better be enabled to exercise upon them the high ministry of our art—the art of curing diseases."

The bibliography of midwifery has greatly increased within a few years past.

"In fact, publication succeeds publication so rapidly, that it is difficult to keep pace with a press so prolific as to fatigue and almost satiate with its excessive productiveness. The obstetric bibliography was oppressive so far back as the days of Mercklin and Linden; and M. Sue, in his curious volumes, exhibits to us a picture of the labours of those writers who, previous to his publication in 1767, had loaded the shelves with essays and tractates, and systems without end. Those old works ought not to be forgotten. Alas! for us,—we live in a degenerate age, when nothing serves us but some newly vamped up exposé of doctrines, which were once refined in the furnace of those intellectual fires, which, if they gave out some dross from their cupels, yet also showed them glowing with much fine gold. I like the enthusiasm of Alex. Massarias, who, having read the works of Hippocrates sixty times, said, iterum vellem legare. And I can almost applaud the old Arabian, who said, se malle cum Galeno errare, quam cum omnibus aliis, bene sentire.

"The works of Hippocrates contain things worth reading; so do those of Galen, those of Celsus, Paul of Egina, Fernel, Mauriceau's work on Midwifery, published in 1668; the pleasing histories of his experience by the Sieur Gillaume Marquis de La Motte, who practised at Valognes, in La Manche, and gave its results to the world in 1715; M. Levret's volume in 1766, those of his great contemporary Wm. Smellie, William Hunter's Anatomy of the Gravid Uterus, Jean Louis Baudelocque's treatise, Gardien's copious disquisitions; the late admirable results of the attention to our subject of one of the finest heads in Europe, I mean that of M. Velpeau, whose system of midwifery, and his treatise on Ovology or Embryology are worthy of his fame; the philosophical disquisitions, and the detailed cases, looking like portraits, by Mad. La Chapelle; Moreau, Chailly, Cazeau, Ollivier, Brierre de Boismont, Montgomery, Collins, Churchill, Rigby, Wagner and Barry. I might present you a long catalogue. All these writers are worthy of your regard. There is not one of them from whom you could not reap a harvest of information, which it will be your pride, your glory, and more, your happiness, to apply for the redemption of the lives that, but for such ministry, were already sacrificed."

We select the following picture of the responsibilities and trials, as well as the consolations of the practitioner of midwifery, as characteristic of the professor's style, and an effective though a somewhat over-wrought sketch.

"How painful are the responsibilities of the art. Here take this case. Suppose one of you, young gentlemen, should graduate here next spring, and settle in some town where you are to be the sole physician. A lady informs you that she is seized with symptoms of labour, and requires your aid. In the morning she had risen from her bed in the most perfect health—the object of a thousand tender interests and ties by which she was bound to that whole society—and by how much closer bonds to her family!

"There is perhaps not one of you who, by reverting to home, cannot single out, in imagination, some lady of the district, who may serve as the eidolon of my theme. Select one then, and accompany me in imagination while I show you what you have to do. You must go—you are the medical man, and you have announced yourself as such. Perhaps you may feel a little qualmish at the thought of what you are to meet. You cannot help but go. Well, you meet her husband at the door. How does he greet you? The other day I returned to my house in the evening. A gentleman was in my office; he had been waiting there for me. He came to call me to his daughter, and desired my presence and attention at the earliest moment. His manner to me was more solemn than if he had come to demand the payment of a thousand pounds.

"When I reached the mansion, I sat for a few moments in the parlour, and the sound of carriage wheels, arrested at the door, was followed by the hurried ring of the bell. It was the young husband just arrived with the nurse. He came into the room and took my hand, which he wrung, while he turned away his head, but did not speak a word, nor could he conceal the tears which were springing from the deep fountains of his emotion, where were welling up such gushing affections as you may suppose of one who saw the jewel of his soul in some possible danger. Now that young gentleman never shook hands with me in his life before. But on this occasion he took it, and seemed to say, 'Sir, I surrender into your hands, the most sacred trust: in doing so, I rely upon you as, next to my Maker, the being to whom I am compelled to appeal in this my extremity.' I was not surprised at this. Gentlemen, had you seen that young lady, more blooming than the rose upon its stem—had you marked the patience with which she bore the unimaginable pangs of the travail—had you witnessed the outpourings of her full soul, as she presented to her father, and her husband, the grandchild and the son, which came to bind them still more closely in the holy community of sentiments, interests, affections, hopes!—you would no longer be surprised at the state of mind which exists under these circumstance. For how shall a man look, without fear, upon the approach of a conflict in which his *peace* may be slain! But let me go on with my story. You proceed to the residence of your patient. She receives you cheerfully, and puts herself under your protection. Yes, the labour is begun. It promises a favourable, and even a speedy issue. It will be over by six o'clock this afternoon. At six o'clock she is still in her agony. What an agony! Twelve o'clock, the progress is slow. The night is fleeing fast—but it is passed in groans, in a thousand vain efforts, vain expectations, vain hopes. She repeats a thousand times in plaintive tones 'I can never bear all this, I shall surely die.' Her friends look at you with inquiring eyes; even suspiciously, and they bend down their faces to ask you, with whispers—Is all right? When will it end? What is your *real* opinion. Meanwhile the progress is slow—but it is progress; I believe. She becomes more restless—more distressed; streams of perspiration are running from her head and breast. The pains are short and feebler—they are separated by longer intervals. Feel her pulse. Good heavens! it beats one hundred and forty in the minute, and her wrist is now cold. The presenting part of her child is *perhaps* advancing—you hope—yes you hope, when you have no hope that the next pain may be a good one; it comes—but it is feebler than the last, and as it goes off you hear her voice—what does she say? I know not—she mutters something! Won't the next pain answer? No! She looks alarmed. Her features are utterly changed. She has lost her comeliness, and they have an unearthly expression. There is brooding over them that shadowy wing, whose rustle you seem almost to hear, preparing to soar upon its long, long flight. Her face is of a bluish cast. Her lips are swollen, and her eyes, which were as doves eyes in the morning, have a dull and unmeaning stare. What did she say again? Strange wanderings! incoherences! She is surely speaking to the phantoms of her far off friends. Compare her as she is with what she was when she saluted you gracefully and modestly in the morning, and invoked your science, your judgment, your skill. Where are they? Is there no agitation for us in such a case!! What are the agitations of commerce, those of the bar, compared with these? Again—the child does not descend—it is evident that it is arrested in the pelvis—by the bony sides of which it is held, as in an iron vice—you cannot make it recede, and it will not advance. Her constitution meanwhile, worn out and exhausted with the vain efforts of the travail, is rapidly passing into the state technically denominated exhaustion, a little progress in which is a little progress towards—*death!*

"That lady, from the far down depths of her misery and danger, calls upon you to rescue her, to save her! Here, too, is a whole great establishment, got up solely upon her account; costly aad magnificent,—the seat of many hopes and great happiness. If you let her die—What did I say? Yes! if you let her die, what is it all worth? Think of those bowed windows, that banner of mourning pendant at the door; the mirrors covered up from the light, as if men should shun the reflection of their own faces. And if you stop at the stair foot you will hear the sound of sobbing, and the convulsive heavings of a strong man's breast. Listen to my lectures. Look at me when I speak to you. Turn not your face away from my demonstrations, and I shall teach you how to overcome all this evil with good—yea, with much good. Take this blessed implement of art, which, like a revelation from heaven, comes so often to lead us by the hand out of places when, but for its merciful interposition, we should be beset with unconquerable difficulties. Take this,—use it aptly; use it just as it ought to be, and the victim is unbound, and the whole crumbling fabric of that household of peace, and joy, and great anticipations of long years of happiness, is redintegrated and restored, and by your masterly hand. If you are a fool, if you are an ignoramus, if you have neglected the opportunities you have enjoyed, you will either fail altogether of the needful skill, or this very instrument will become the means still more surely of showing you that beautiful woman, who was committed to your care but a few hours ago, in perfect health, and vigour, and glory, a ghastly corpse, with the pale face of its dead baby lying by its side. Now, how much does it cost a man before he can become nearly callous and indifferent to the prospect of such scenes as I have sketched for you? How long before he can learn to say, while all around is panic, and terror, and wild apprehension, with his calm soul unshaken by the moral whirlwind that rages around him?—Silence—be still—wait. Trust in *me;* and on—and still on he ventures, until, seeing that the fit moment is come, he takes in his hand this beautiful instrument of pure mercy, and power, and beyond the reach of sense or sound, he adjusts it speedily, noiselessly, without the least pain or danger, and by a few masterly movements of his hand—lo! that delicate creature, which, as Melancthon says, was *matricida sed moriturus*, beyond all hope, opens its glad and gladdening eyes to the light of the day. And then! who can pour out like her, like that new made mother, the rich effusions of a heart that rebounds from the borders of the grave, and sings and soars far, far up, up, and up to the very heaven of the holiest emotions, of gratitude to God for release from mortal pain, and for redemption from death itself. Look at that household! Is there a scene in the whole circle of the world where two human voices can be lifted up to say, with greater unction, Behold,we have the oil of joy for mourning, and the garment of praise for the spirit of heaviness!

"There remains one trial more: it is when the surgeon fearfully casts his eye upon that child, in order to discover whether possibly some trace of the blade may have been left upon its form. It is a moment of intense anxiety, fully compensated by finding that it lives, and moves, and has its being, without spot or blemish, or the least impression of his daring operation. And the mother! it is well with her too.

"Such, and frequent are the scenes through which you are destined to pass, young gentlemen! I do not pretend that I, or that any man can teach you how always to bring to such fortunate issue, the heavy charge of the happiness of families. Nor can society, nor will it, indeed, expect so much at your hands. But this it will expect. It will expect that you should know of no embarrassment in the discharge of your duty; that you should be able to say this is right, or that is wrong. What a great, and what an abiding consolation, for a man to be able clearly to discern what is due to himself and and others on such occasions; to enjoy a perfect confidence in the knowledge of his art! But, on the other hand, how must he feel, who knows he has forfeited not only the life that was, humanly speaking, put into his keeping, but in doing so, has lost with it his own self-respect.

"I cannot but think, gentlemen, that I try your feelings upon this occasion. I cannot suppose that you are callous enough to look on such a picture as I have drawn, and which comes not near to the sober truth, for no tongue can tell it, (I appeal to the many physicians here to say if it is over drawn,) without feeling in some degree, that if what I have represented be even like the truth, it will be necessary for you to do your duty, as far, at least, as it is connected with the particular department of instruction that is under my charge. I hope I have tried your feelings. I wish I could harrow them. I wish I could make you sensible in your heart of hearts, of the pangs I have endured on such occasions; pangs that might have been spared me, had I known in my early day what I have since been *obliged* to learn. Thank God! that lesson I have learned long ago."

